# Influence of the pandemic dissemination of COVID-19 on radiotherapy practice: A flash survey in Germany, Austria and Switzerland

**DOI:** 10.1371/journal.pone.0233330

**Published:** 2020-05-21

**Authors:** Melanie Reuter-Oppermann, Ralf Müller-Polyzou, Holger Wirtz, Anthimos Georgiadis

**Affiliations:** 1 Fachgebiet Wirtschaftsinformatik | Software & Digital Business, Technical University of Darmstadt, Darmstadt, Germany; 2 Institut für Produkt- und Prozessinnovation, Leuphana Universität Lüneburg, Lüneburg, Germany; 3 Gemeinschaftspraxis für Strahlentherapie Singen-Friedrichshafen, Singen, Germany; MD Anderson Cancer Center, UNITED STATES

## Abstract

**Background:**

The COVID-19 pandemic has already changed our globalised world and its long-term impact is not yet known. It is apparent that businesses and institutions are increasingly affected. COVID-19 discussions often focus on intensive care units in hospitals. However, COVID-19 also effects life-saving and -prolonging radiotherapy for patients suffering from cancer.

**Method:**

We have conducted a structured online survey among medical physicists in Germany, Austria and Switzerland from March 23rd to 26th 2020. In total 154 responses (82 completed, 72 partially completed) were analysed in the context of the COVID-19 dissemination.

**Results:**

72.4% of the respondent’s state that their processes are affected due to COVID-19, while the top three answers are longer processing times (54.2%), patient no-shows (42.5%) and staff reduction (36.7%). 75.8% expect further unavailability of their personnel in the upcoming weeks. All participants have already taken several measures, especially providing information for patients at the entrance (89.6%) or over the phone (73.6%), restricting access for accompanying persons (77.4%) and providing disinfectant at the entrance (72.6%).

**Discussion:**

The results presented in this article aim to support business continuity and risk management for radiotherapy centres to prepare for future challenges. The results show that most radiotherapy centres has implemented initial contingency measures, applying them pragmatically. The main problem however remains, that is the high risk of infection both for patients and medical personnel along with the associated risk of temporarily loss of personnel and ordered closure of business.

## Introduction

A pneumonia of unknown cause was first reported in Wuhan China on December 31st, 2019. Within one month the outbreak was declared a Public Health Emergency of International Concern. It took until March 11^th^ that COVID-19 was recognized a pandemic by the World Health Organization (WHO) [1]. The first case of COVID-19 in Germany was reported on January 27^th^ [2]. The situation worsened daily and on March 19^th^ the Federal Chancellor of Germany Angela Merkel addressed the nation: “…since the Second World War, there has not been a challenge for our country in which action in a spirit of solidarity on our part was so important.” [3]. According to the World Health Organization, on March 22nd, Germany had already 21.463 confirmed COVID-19 cases with 67 deaths. Austria reported 3.024 cases with 8 death and Switzerland 6.077 with 56 deaths. From March 22nd to 26th, and thus during the period of the survey, confirmed COVID-19 cases in the three countries increased by 70.5% to a total of 52,110 [4]. Prior to this the International Labour Organization issued a note on the impact of COVID-19 on the world of work [5]. Also first collected clinical experience from healthcare workers in China was already shared [6]. For Germany, the Robert Koch Institute (RKI) is the government’s central scientific institution that monitors the national COVID-19 situation and estimates the corresponding risk for the population [7]. Within only five days between March 21^st^ and 25^th^ numerous far-reaching measures were decided and implemented by the federal Governments of Germany, among them assembly bans and ordered business closings. Information on the COVID-19 virus and rules of conduct for patients were published by the Federal Ministry of Health [8]. On March 13^th^ the president of the European Federation of Organisations for Medical Physics (EFOMP) described COVID-19 as an unprecedented challenge to European health systems and outlined the role medical physicists have to play in helping cancer patients and staff in this situation. Additionally, first recommendations were published by EFOMP [9]. The professional association for radiotherapy ARO and DEGRO released a statement just before the start of this research declaring that the spread of COVID-19 in Germany is expected to have a major impact on radiotherapy [10]. A second statement published by DEGRO on March 25th provides initial information on actions radiotherapy departments can take in relation to their workforce [11]. The association expected that within weeks or months patients or employees will be infected and technical service for radiation equipment will be impaired while cancer patients depend on an effective and timely treatment. Therefore, there was a great need to promptly record and evaluate the current and planned measures in radiation therapy centres.

The aim of this paper is to evaluate how radiotherapy centres assess the COVID-19 situation. The understanding of the technical and non-technical situation of the radiotherapy centres is a prerequisite in order to analyse the business contingency and risk management measures already applied, evaluate the process impact and compare potential additional mitigation actions. We have conducted a structured online survey among medical physicists in Germany, Austria and Switzerland from March 23rd to 26th 2020. This was at a time when measures for radiotherapy were not yet comprehensively and structurally communicated but the number of confirmed cases in all three countries increased fast. In total 154 responses (82 completed, 72 partially completed) were analysed in the context of the COVID-19 dissemination. In this critical situation, the survey reveals procedural weaknesses in an extraordinary stress situation and thereby offers the opportunity to identifies new research questions. Overall, the survey aims to support the development of business continuity and risk analysis knowledge in radiotherapy. The domain knowledge will serve as a basis for future research projects improving radiotherapy performance through operational efficiency and risk reducing measures as well as the design of intelligent solutions.

The paper is structured as follows: In the following section, we summarise the current literature on COVID preparedness and countermeasures for radiotherapy incidents. Then, we present the study design and display the results, followed by a discussion. Based on the results and the existing literature, we propose a research agenda with topics for future research. We finish the paper with a summary and conclusions.

## Foundations

In several countries researchers and practitioners have published initial guidelines for radiotherapy during the COVID-19 crisis. With a focus on the US, especially on the Seattle Cancer Care Alliance, Fred Hutchinson Cancer Research Center, and University of Washington, Ueda et al. discuss actions in radiotherapy and potentially difficult decisions about how and when providing cancer treatment becomes a necessity [12]. Their aim is to provide cancer treatment to patients in need, as safely and as justly as possible. The Yale School of Medicine Department of Therapeutic Radiology faculty has also created guidelines for the COVID-19 crisis [13]. They include several flow charts and the RADS framework by Zaorsky et al. RADS stands for “Remote visits, Avoid radiation, Defer radiation, Shorten radiation” [14]. Mossa-Bassa et al. present COVID-19 preparedness measures for radiology departments in several US hospitals [15]. The National Health Service (NHS) has published a guidance for infection prevention and control in nuclear medicine during COVID-19. It presents three patient classes, for example, red, amber and green, to distinguish if patient appointments should be cancelled or rescheduled [16]. Motivated by the heavy outbreak in Italy, Filippi et al. present practical indications for radiotherapy departments. They define five claims: (1) radiation therapy must be ensured for cancer patients (2) safety of health professionals, patients and caregivers must be ensured (3) protocols are necessary for handling of COVID-19 suspects or positive patients (4) staff must be reorganised (5) patients’ access to radiotherapy clinics must be limited [17]. Krengli et al. summarise the operation plans that were established in a radiation oncology department of a hospital in Novara, Piedmont, Italy [18]. Simcock et al. summarise the results of a #radonc community online meeting with 121 contributors from 17 countries and 6 continents on topics around infection prevention, rationalisation of workload and working practice in the presence of infection [19]. The European Society for Radiotherapy and Oncology (ESTRO) has published a list of advices for European medical physicists in hospitals and clinics [20].

When looking more closely at the guidelines and measures, three main categories can be identified: “patients”, “processes” and “personnel”.

Yu et al. investigate the COVID risk for cancer patients in Wuhan, China. They found an elevated risk for cancer patients catching the virus and therefore suggest proper isolation protocols for those patients who require treatment [21]. Liang et al. present a nationwide analysis of COVID infection risks for cancer patients in China. They found a higher risk of severe events for cancer patients [22]. While several publications state guidelines for radiotherapy centres during COVID-19, to the best of our knowledge studies are still missing on how radiotherapy centres actually reacted to the virus, especially in Germany, Austria and Switzerland, if they have implemented the guidelines and if it was easy/ possible to implement them. The aim of our research is to start closing that gap.

## Study design

An online survey was conducted using an email distribution list of medical physicists in Germany, Austria and Switzerland. The study was designed and implemented in LimeSurvey [23]. The questionnaire for the online survey was reviewed by a radiotherapy expert and extensively tested. Overall, the survey was optimised for high acceptance according to Schnell et al. [24]. Special effort was spent on the welcome message, the duration to complete the questionnaire and the formulation of questions and answers. At the beginning of the questionnaire, the participants were informed about the specific background of the survey and that the results would be used for scientific purpose by the researchers from the two managing universities of Darmstadt and Lüneburg, Germany. The required time to complete the questionnaire was clearly communicated. In addition, the anonymity of the answers was pointed out. The announced timely communication of the survey results served as a motivation factor. Most questions were formulated as closed-ended or semi-open questions for greater objectivity. The answers were either non-mandatory or an "I don't know" option was provided in order to avoid that the interviewees felt urged to a specific answer. Before submitting the results, the participants had the opportunity to leave further comments. At the end of the questionnaire contact details for queries about the questionnaire and scientific results were displayed. The survey questionnaire can be downloaded at S1 File.

### Sample group and implementation

This empiric study focuses on medical physicists. Medical physicists are familiar with the technical environment, the radiotherapy workflows and interact with the personnel due to their safety and quality responsibilities. Medical physicists are therefore particularly suitable for the purpose of this study. The survey invitation was sent to the Email list MedPhys-D maintained by the German Society for Medical Physics (DGMP) [25]. The German MedPhys-D mailing list is operated by the Clinic for Radiotherapy at the University of Würzburg, Germany. It is available to DGMP members and those interested in medical physics. It provides a fast and uncomplicated way to exchange information. The invitation was also sent to the members of the working group Risk Management of the DGMP [26]. In total, 82 participants in Germany and Austria fully completed the questionnaire, while 72 participants have partially answered the questions.

A high number of participants was expected due to the topicality of the subject matter. The survey was accessible for a period of four days, from Monday 23rd of March to Thursday 26th of March. The survey was conducted in German. More than 40 participants completed the questionnaire already during the first day. IBM SPSS Statistics Ver. 26 was used for data analysis [27].

### Questionnaire structure

The questionnaire consists of nine sections with 15 questions as displayed in Table 1. It includes closed-ended questions with “Don't know” and “Others” options, questions with multiple selection options, and one open-ended question. A few questions use a 5-Likert scale, also with “Don't know” and “Others” options.

**Table 1:** Structure of the questionnaire.

Section	Questions	Background
1. Welcome and introduction		Information and motivation
2. Basic information	5	Sample analysis
3. Effects of COVID-19 on radiotherapy	3	Situational analysis
4. Measures against COVID-19	1	Situational analysis
5. Process impact of COVID-19	1	Impact analysis
6. Contingency Plan and measures	3	Preparation analysis
7. Potential restrictions on operation	1	Implementation analysis
8. Recommendations or suggestions	1	Ideation
9. Thank you message		Information

The questionnaire is framed by Section 1 which contains the introduction page with a welcome message, and Section 9 which contains the appreciation message and provides contact details. Section 2 collects basic information about the participating radiotherapy centres and thereby supports the sample analysis. The questions refer to the country of operation, the type of radiotherapy centre, the number of locations, the number of linear accelerators (linacs) in operation, the number of treated outpatient/ inpatients per year and the number of treated benign/ malign tumours.

Sections 3 and 4 analyse the current situation in the radiotherapy centres. It is investigated to what extend the overall processes in radiotherapy are affected by COVID-19. It is followed by two questions to understand better how the radiotherapy centres are currently affected. The situational analysis is concluded by a question focusing on restrictions that the participants expect in the coming weeks and which corresponding measures have already been taken or are planned. The impact analysis of Section 5 evaluates how the participants perceive the influence of COVID-19 on the specific processes in their radiotherapy centre. Section 6 contains three questions related to contingency plans and related measures. The goal is to understand the level of preparation for the expected dissemination of COVID-19. Section 7 collects further implementation information. Participants are specifically asked for measures that would be taken in case the operation of the radiotherapy centre had to be further restricted. Section 8 consists of an open-ended question to collect potential further ideas from the participants.

## Results

As stated above, we received 82 complete questionnaires, while 72 participants partially answered the questionnaire. We decided to use all answers given to maximise the potential gain of knowledge, while being careful with the conclusions that can be drawn from the data. We will therefore state the number of responses (n) for each question.

### Basic information

According to the IAEA DIrectory of RAdiotherapy Centres (DIRAC) there are currently 286 radiotherapy centres in Germany, 16 in Austria and 36 in Switzerland that operate 523, 49 and 71 linacs [28]. DIRAC information is provided voluntary and does not include all radiotherapy centres available. Corrected figures provided by experts are 292 centres in Germany, 16 in Austria and 42 in Switzerland, out of which 25 are located in German speaking regions [29].

Table 2 presents the correlation between country, type of radiotherapy centre, number of patients and number of linacs in the set of respondents. Note that only 43 respondents stated all the numbers of stationary and ambulant treatments of benign and malign tumours per year. They are therefore probably not representative for the sample group. For the first four columns, an entry x (y) means that out of the overall 150 respondents x chose this answer and y belonged to the 43 respondents that also stated the numbers for the other 4 columns.

**Table 2:** Correlation between country, type of radiotherapy centre and number of linacs.

n = 150 (n = 43)	One linac	Two linacs	Three linacs	Others (≥4)	Ambulant patients	Stationary patients	Benign tumours	Malign tumours
Respondents from Germany	11	60 (15)	31 (8)	41 (17)	1352	375	448	1301
Respondents from Austria	0	1 (0)	0	4 (2)	2050	1100	225	2175
Respondents from Switzerland	0	2 (1)	1 (0)	0	600	100	40	660
Part of public hospital	6 (0)	30 (10)	21 (5)	29 (15)	1231	475	362	1351
Part of a private hospital	0	6 (1)	2 (1)	1 (0)	1150	215	180	1035
A private radiotherapy centre	4 (0)	23 (2)	9 (2)	14 (4)	2071	275	778	1501
Others	1 (0)	4 (3)	0	0	990	143	313	813
Total / average	11	63	32	44	1964	470	662	1726

56.2% of the respondents (n = 153) state that their radiotherapy centre is part of a public hospital, 5.9% are part of a private hospital and 33.3% are private radiotherapy centres. 4.6% of the respondents choose “Others” including four ambulatory healthcare centres (German: MVZ) which are interdisciplinary, physician-led facilities designed to provide interdisciplinary care from a single source through structured cooperation [30].

60% of the respondents (n = 150) operate at one location, 24.7% at two locations and 6% at three locations. 9.3% of the respondents choose “Others” including four times four locations and five times more than five locations. 7.3% of the respondents choose that they operate one linac, 42% two linacs, 21.3% three linacs, 12% four linacs. 15.4% state that they operate more than four linacs, 2% did not give any number. Therefore, most of the respondents operate two or more linacs, half of them even 3 or more, which might increase the possibilities to counteract on COVID-19.

The majority of patients are treated ambulatory and for malign tumours. Nevertheless, many respondents state a comparably high number of patients with benign tumours. As stated in Filippi et al., the treatment of malign tumours should have higher priority compared to benign tumours during the COVID-19 crisis [17]. Reducing the treatment of benign tumours would free up time that could then be used for additional safety measures against COVID-19, for example.

### Processes and expected restrictions

72.4% of the respondents (n = 112) agree that the processes of their radiotherapy centres are affected by COVID-19 to some extent, as shown in Table 3. That means that the COVID-19 pandemic has already an impact on the radiotherapy centres, even though none of the respondents reported COVID-19 patients at the time of the survey. While the cumulated probabilities for public hospitals and private radiotherapy centres are similar, a higher percentage of private centres state that they are affected. Linking the responses with the numbers of linacs clearly shows that larger clinics are more affected. The corresponding Table 3A and 3B can be found in the S2 File.

**Table 3:** The processes in my radiation therapy are affected by COVID-19.

n = 112	Frequency	%	Cumulative %
Affected	38	34	(affected) 72.4
Mainly affected	17	15.2	
Partially affected	26	23.2	
Mainly not affected	22	19.6	(not affected) 25.8
Not affected	7	6.2	
Not specified	2	1.8	(not specified) 1.8

Furthermore, the participants were asked by which factors the processes in their radiotherapy department are affected. Table 4 shows that 54.2% of the respondents choose that processes take longer due to introduced protective measures. 36.7% of the respondents claim that they work with reduced personnel. That 42.5% of the respondents answer that patients do not appear for appointments is an alarming result, as interrupted therapies can potentially have negative consequences and create additional work for various employees in the radiotherapy centre. The combination of increased time spent on protective measures with reduced numbers of staff could indicate a higher workload for the remaining personnel. When linking the responses to the facility types, it becomes apparent that their processes are affected differently. More than half of respondents working in public hospitals reported longer processes due to protective measures (54.2%). No-show rates, staff reduction and limited access were all similarly affected and chosen by around a third of the respondents. In private centres longer processes due to protective measures and patient no-shows are the main issues (57.9%), In addition, the staff reduction and the effects on the supply chain seem to be more severe than for the other facility types. All numbers can be found in the S2 File.

**Table 4:** Processes in radiotherapy affected by COVID-19.

Answers (n = 120, multiple selection)	Frequency	%
Longer processes due to protective measures	65	54.2
Patients do not appear for appointments	51	42.5
Reduced own personnel	44	36.7
Limited access possibilities (traffic)	30	25
Supply chain affected	25	20.8
Reduced own personnel at co-handlers	14	11.7
Failure due to missing equipment service personnel	13	10.8
Others	23	19.2

A total of 23 participants selected the option “Others”. The corresponding answers were analysed manually and matched with to the three main categories for guidelines and countermeasures identified in the literature, “patients”, “personnel” and “processes” (Table 5). The number of mentions was neglected in this process. It was also reported by one participant that patients from other radiotherapy centres have forwarded patients, even though they were not tested positive on COVID-19. This could be an indicator for the current pressure on radiotherapy centres.

**Table 5:** What are the processes in your radiation therapy affected by? [Others].

Patient	Personnel	Processes
Patient appointments for benign tumours cancelled or postponed	Time consuming protection measures for personnel	Patients are late because they have to wait in queues
Not urgent treatments are cancelled	No accompanying personnel allowed	Limited seating
No aftercare for benign patients	Personnel at home as backup	One linac only for COVID-19 patients
Apply strict indications for malign patients	Reduced personnel/ smaller teams onsite	No technical maintenance services
Aftercare only via telephone	Weekly shifts only	Deduction of equipment because hospital prepares for COVID-19 emergency
	Employees partially in home office	

In the next question, the participants were asked about the restrictions they expect for the upcoming weeks. The results are summarised in Table 6. Overall, the participants expect further restrictions. The number one concern of the participants is the non-availability of own personnel in the future with 75.8% (+39.1 percent points). Increasingly longer processes due to protective measures are expected (+4.1 percent points), as well as more patients who will not keep their appointments (+14.2 percent points). Also, the mentions of person related restrictions of “non-availability of personnel at co-handlers” and “failure due to lack of access to service personnel” increase by 32.5 percent points, respectively 31.7 percent points.

**Table 6:** Restrictions expected in the coming weeks.

Answers (n = 120, multiple selection)	Frequency	%
Non-availability of own personnel	91	75.8
Longer processes due to protective measures	70	58.3
Patients do not keep appointments	68	56.7
Non-availability of personnel at co-handlers	53	44.2
Failure due to lack of access to service personnel	51	42.5
Supply chain affected	35	29.2
Limit access possibilities due to transport situation	33	27.5
Others	12	10

The 12 “Others” answers are related to patients and personnel. The treatment of COVID-19 infected patients is expected, as well as a reduction of patients due to staff shortage. Therefore, more appointments are expected to be cancelled. On top of this, loss of personnel due to home office and high workload is expected. One participant even stated that personnel will be pulled out of the radiotherapy department in order to support wards with COVID-19 patients.

Linking the responses to the facility types shows that all facility types rate the non-availability of own personnel highest. While public and private hospitals expect more restrictions due to longer processes, private centres expect less future restrictions. With one out of three respondents from public hospital stating limited access possibilities in transport is going to be an issue in the upcoming weeks, it is a significantly bigger issue than for the other facility types. Private centres and hospitals expect more patient now-shows than public hospitals. Detailed numbers can be found in the S2 File.

Afterwards, the participants were asked about measures that have already been taken by radiotherapy centres. The results are shown in Table 7. The participants have already implemented a variety of measures. These measures are mainly related to information policy, access restrictions and hygiene measures, such as disinfection stands and seating patients with larger distance. Some measures, e.g. home office work, were also encouraged by the public news coverage as shown in S3 File. Performing tumour boards in video conferences and separating work teams has only been implemented by 31.1% respectively 30.2% of the respondents.

**Table 7:** Measures already taken.

Answers (n = 106, multiple selection)	Frequency	%
Information for patients at the entrance	95	89.6
Access restrictions for accompanying persons	82	77.4
Information for patients in telephone contact	78	73.6
Disinfection stand at the entrance	77	72.6
Changed seating concept, e.g. waiting area	76	71.7
Reduction of meetings	74	69.8
Access restrictions for taxi drivers	67	63.2
Access restrictions accompanying children and young people	65	61.3
Reduced physical contact with patients	61	57.6
Greater distance between employee seats	61	57.6
Hygiene training for all employees	55	51.9
Home office for selected employees	55	51.9
Use of standardised questions in case of suspected corona	50	47.2
Information for patients on the website	45	42.5
Employee childcare	36	34.0
Tumour board as video conference	33	31.1
Separating employees in teams	32	30.2
Bringing forward measures such as technical maintenance or QA	12	11.3
Others	15	14.2

The 15 “Others” answers are again categorised into “patient”, “personnel” and “processes” as presented in Table 8. One participant proposed patient communication via Skype or the smartphone in general. An example of a COVID-19 application for patients is presented by the Charite University hospital [31]. Such a telecommunication based service has already been described for remote tumour board discussions.

**Table 8:** What measures have you already taken? [Others].

Patient	Personnel	Processes
Patient communication using Skype or Smartphone	Distribution of protection masks for personnel with the obligation to wear them	Regular disinfection of the registration and the patient waiting area
Cancelation of aftercare appointments	Ordered leave of absence	Tumour board with reduced participants and via telephone
Distribution of protection masks for patients with the obligation to wear them	Separate work teams	Dedicated linac for COVID-19 patients
		Delay of SV quality check

The radiotherapy therapy process is based on a number of process steps in the different work areas such as patient handling, imaging, planning, treatment and aftercare. In the following, we have analysed how the survey participants foresee the COVID-19 impact on the process steps of radiotherapy. The results are presented in Table 9.

**Table 9:** Expected impact of COVID-19 on process steps of radiotherapy.

Answers (n = 88)	Strongly agree (%)	Agree (%)	Undecided (%)	Disagree (%)	Strongly disagree (%)	Don't know (%)	N/A (%)
Appointment planning	**32.6**	28.1	**32.6**	5.6	2.2	0.0	7.9
Aftercare	25.8	**34.8**	11.2	6.7	1.1	7.9	12.4
Tumour conference	16.9	**34.8**	12.4	11.2	2.2	12.4	10.1
Anamnesis, examination, discussion	**25.8**	24.7	24.7	6.7	4.5	4.5	9.0
Patient decision making	9.0	**33.7**	22.5	11.2	2.2	7.9	13.5
Visits and final examination	6.7	**36.0**	23.6	13.5	2.2	7.9	10.1
Collection of patient data at admission	13.5	18.0	**28.1**	23.6	3.4	3.4	10.1
Imaging	3.4	16.9	18.0	**25.8**	20.2	5.6	10.1
Immobilisation for treatment	4.5	14.6	21.3	**23.6**	20.2	6.7	9.0
Patient verification	5.6	5.6	20.2	25.8	**29.2**	4.5	9.0
Treatment planning	1.1	9.0	18.0	**37.1**	25.8	1.1	7.9

The answers were additionally grouped according to the agreement values (strongly agree plus agree). The lowest impact is expected for the process steps patient verification, immobilisation, imaging and treatment planning. The agreement reaches values above 40% with the increased patient interaction for anamnesis, visits, examinations and patient decision making processes. Aftercare and appointment planning are expected to be most affected by COVID-19 reaching agreement values above 60%.

It becomes apparent that public hospitals expect a higher impact on imaging than private facilities. One reason could be that they often have to share resources as CTs and MRTs with other departments. Private facilities usually have full control about their own resources. While private centres do not expect difficulties with patient verification, 9.1% of the respondents from public hospitals do. We assume that higher staff and patient numbers together with larger buildings could be a reason, which is actually independent of COVID-19 and might therefore be a general issue.

### Failure concept and contingency plan

Operators of radiotherapy centres in Germany have to provide a technical failure concept to the authorities to ensure that patient treatment is not interrupted [32]. The majority of the respondents (63.9%, n = 83) state that their contingency plan is based on a tandem concept with two identical or technically compatible linacs. 25.3% operate several linacs, which are technically not equal. 18.1% rely on contracts with cooperation partners in geographical proximity.

Subsequently, the participants were asked about the measures of their contingency plan that they had to implement already and that they expect to implement in the future. The results are presented in Table 10. Classification of patients into urgency groups was implemented by 41% of the respondents. Further implemented measures are treatment related, such as hypofractionation (33.7%), recalculation of treatment plans for equivalent continuation of treatment (18.1%) and compensation of treatment breaks with additional fractions (18.1%).

**Table 10:** Measures of contingency plans already implemented and future implementations.

Answers (n = 83, multiple selection)	Already implemented		Future implementation	
	Frequency	%	Frequency	%
Classification of patients into urgency groups	34	41.0	51	61.5
Treatment with hypofractionation	28	33.7	40	48.2
Recalculation of the treatment plans for equivalent continuation of treatment	15	18.1	38	45.8
Compensation of treatment breaks through additional fractions	12	14.5	33	39.8
Treatment with fractions during the weekend	7	8.4	20	24.1
Planning of alternative treatment plans for different linacs	7	8.4	13	15.7
Use of linac capacity of cooperation partners	1	1.2	10	12.1
Replanning from particle to photon beam therapy	0	0	2	2.4
Others	12	14.5	3	3.6

The 12 respondents that chose the option “Others” state that they did not have to implement any measures of their contingency plan however one respondent states that “[…] treatment during the weekends is unlikely, because the bottleneck is the personnel”.

In the future, radiotherapy centres would first extend the existing measures. The classification of patients into urgency groups increases by 20.5 percent points to 61.5%. Hypofractionation would be implemented by 48.2% of the respondents. This reflects an increase of 14.5 percent points. The compensation of treatments trough additional fractions would be implemented by 45.8% of the respondents. Shift operation at the radiotherapy centre was mentioned by one participant as an additional measure that could be implemented.

Finally, Table 11 shows what measures the participants would first implement if the treatment had to be restricted. The participants would first postpone or not perform treatment of benign tumours. Also, aftercare examinations would be postponed. Treatment with hypofractionation is considered by 58.5% of the respondents. Postponement of defined treatments by a few weeks seems to be less accepted (26.8%). Postponement of palliative and adjuvant radiotherapy were only chosen by 14.6% and 11%. In the category “Others” participants additionally mentioned aftercare via telephone and the admission limitations for new tumour patients.

**Table 11:** Measures that would be implemented if the treatment had to be restricted.

Answers (n = 82, multiple selection)	Frequency	%
Postponing the treatment of benign tumours	73	89.0
No treatment of benign tumours	54	65.9
Postponement of aftercare—examinations	51	62.2
Treatment with hypofractionation	48	58.5
Postponement of defined treatments by a few weeks	22	26.8
Postponement of palliative radiotherapy	12	14.6
Postponement of adjuvant radiotherapy	9	11.0
Others	4	4.9

## Further suggestions

Concluding the survey and being asked for comments or suggestions the participants describe the current situation as follows:

“At the moment we are driving on sight, some things are already in the pipeline, but what is still possible/necessary we have to decide situationally. At the end, the public health department also plays an important role, especially if we will have infections of the staff or/and the patients […]”

“Our ward is already being emptied as much as possible in order to be able to accept patients from other wards, which in turn need capacity for Covid-19 patients.”

“Unfortunately, the physicists are sitting in a six-man office. Medical-technical assistants are in groups at the linac. The clinic management sees no need for weekly teams to ensure operation and reduce the risk of infection.”

“Very high procurement problems for protective clothing and hand disinfectants, which will influence the treatment processes at some point in time.”

The participants foresee:

“Extreme effort for the treatment of infected curative tumour patients.”

“Our concept would provide a similar procedure for the treatment of symptom-free COVID patients as for MRSA, Norovirus, clostridia etc. patients. Patients pause their treatment if they have fever, cough, or other infections.”

“The risk of COVID-19 is estimated to be low as it is a potential threat. However, our patients are really threatened by their cancer. All possible protective measures are taken to prevent its spread. Nevertheless, the treatment of our patients is the main focus, as otherwise they will actually die from their existing disease.”

Some participants propose additional measures for the formation of working groups. They suggest to create teams in the different professions alternating every two weeks so that only half of the employees are on site on a weekly basis. Furthermore, distance measures are mentioned to reduce number of employees and keep distance at the workplace. Looking into the future, the use of risk management groups and IT solutions for risk management is proposed. Finally, the participants conclude: “A common procedure would be desirable including a statement by the German Society for Medical Physics e.V. (DGMP) and German Society for Radiooncology e.V. (DEGRO)”.

## Discussion

The aim of our research was to provide information on how COVID-19 effects life-saving and -prolonging radiotherapy. We wanted to understand how radiotherapy centres reacted to the virus, especially in Germany, Austria and Switzerland, if they have implemented guidelines or measures. Most radiotherapy centres in Germany, Austria and Switzerland are affected by COVID-19. The reported challenges are: 1) longer process times, 2) patients not appearing for appointments and 3) reduced own personnel correspond to the categories 1) processes, 2) patients and 3) personnel. The three areas are related to each other. Patients, who do not keep appointments for instance, generate additional workload due to rescheduling of appointments and possibly even the need for calculating new radiotherapy plans. This is a situation that will become more evident with the expected loss of personnel in the coming weeks. Accordingly, the German Association for Radiooncology has formalised its request towards the authorities to reduce the minimum number of skilled personnel required for linacs [11]. The measures taken against COVID-19 are 1) information policy, 2) access restrictions and 3) hygiene measures. They are consistent with the radiotherapy guidelines developed based on the experience from other countries. Measures enabled by digital technology such as video-based tumour boards and home office work are not yet widely used. The situational analysis conducted in sections 3 and 4 outlines the reality in a distinct way. It reflects the situation in the health sector as described by the German Hospital Association that defines the COVID-19 epidemic as an unprecedented challenge in material and personnel [33]. The impact analysis of section 5 shows that the processes with high patient interaction are most affected by COVID-19. The appointment planning process could be realised fully digital and the patient decision making process could be supported for instance by video-based services [34]. Visits, examinations and aftercare however require higher patient interaction. These processes could be supported by digital solutions such as remote monitoring through collection of patient-reported outcomes [35]. Telemedicine solutions could potentially reduce the number of physical meetings needed but defined success factors must be considered when designing such solutions [36]. The preparation analysis of section 6 shows that most radiotherapy centres are well prepared for technical contingency. The tandem concept is the preferred solution for the mitigation of technical failures of linacs. To address the COVID-19 challenge additional capacity increasing measures and increased flexibility are needed. Hypofractionation increases linac capacity. Continuation of treatment at different linacs or compensation of treatment breaks through additional fractions requires fast, efficient and safe planning. The therapy planning and sometimes related patient quality assurance performed by medical physicists is largely software based and can also be performed from home, provided that secure remote data connectivity to the central computers is available. The classification of patients into urgency groups is the most popular standard measure for radiotherapy centres. This is also stated in the guidelines and concepts summarised in the foundations section. This approach would be continued in case of further restrictions of the operations. Patients with benign tumours would be postponed or not treated at all. Aftercare and new treatments would be postponed, while hypofractionation would be used increasingly. In order to assist radio oncologists in making such ethical decisions, clear and legally secured catalogues of criteria should be developed. Such criteria and corresponding guidelines should be internationally harmonized. This would prepare for future challenges and would facilitate knowledge sharing. While missing face masks and other equipment for staff and patient protection is a big issue in the news coverage, it was not mentioned in the responses. Questions on the availability, the costs and the necessity of protection equipment should be integrated in future surveys. Revised business continuity and risk management concepts should, apart from the technical availability, also consider non-technical factors such as personnel and material supply.

A digitalisation study among medical physicists in Germany revealed that only 30% of the radiotherapy centres have a digital risk management system [37]. That is a fact that, in times of COVID-19, may be an explanation for the individual approach to problem solving. What serves to increase efficiency in normal times can result in reliability in times of crisis. The automation of services should therefore in the future also be evaluated under risk management aspects. The digitalisation study also revealed that only 16% of the centres have digital interfaces to patients and 30% to referring physicians. Mobile applications for digital anamnesis and appointment management were described. The expectations on increased efficiency and error reduction were high, but most likely nobody thought that such applications could add value during a pandemic. Additional advantages were described for the secure integration with referring physicians. Often data must still be transferred manually requiring unnecessary physical contact. Summarising, digitised radiotherapy centre in times of COVID-19 crisis seem to have more opportunities to act rather than to react. Radiotherapy centres should therefore accelerate digitisation strategies to be better prepared for future challenges.

## Limitations

The research study was conducted on a national level in Germany, Austria and Switzerland. The study was conducted in German language, potentially excluding 35% French and 5% Italian speaking radiotherapy centres in Switzerland. The percentage of participants from German-speaking Switzerland was approximately 20 percent points lower than in Germany and Austria. The random sample is probably not representative for the total population, although the processes and work roles in radiotherapy are largely similar in the core areas. Furthermore, the research study was limited to the occupational group of medical physicists. Other occupational groups in radiotherapy might provide more detail on certain research items. The implementation of the study in other countries could lead to different results due to a different COVID-19 situation, the force of different national protective measures or cultural differences. Additionally, research results must be interpreted in the context of the temporal COVID-19 situation. Finally, even though medical physics are usually well coordinated in their teams, it cannot be excluded that more than one medical physicist of one centre participated in the survey.

## Research agenda

The COVID-19 experiences will have a lasting influence on radiotherapy practice. The study presented in this document can therefore only be a first step of a more comprehensive investigation. It is planned to supplement the results obtained with further quantitative and qualitative surveys and analyses. In particular, the results derived from this survey are planned to be discussed in a forum of medical physicists and industry experts. Future research should support the generation and distribution of knowledge to support the development of intelligent assistance systems for physicians, medical physicists, medical technical assistance and cancer patients. In addition to increasing efficiency and reducing errors, the integration into a comprehensive risk management system should be considered. In the following, we list and describe five topics.

### 1. Digitalisation

Digital solutions such as video calls with patients or referring doctors or for tumour boards discussed above should be analysed, evaluated and designed in detail considering the specific requirements of the radiotherapy environment. Such solutions can also be envisioned for advanced patient positioning or workflow solutions with reduced physical contact to patients. A future study could investigate the activities that could be managed remotely and then propose possible solutions, for instance using augmented reality or other available technologies.

### 2. Appointment planning and staff scheduling

Appointment planning and staff scheduling are two well-established operations research topics. Future research could propose models and approaches to schedule patients and staff considering the increasing patient no-show probabilities, potential sickness of staff and the possibility that they might be infected or are infected. For both planning problems, staff schedules and appointment planning, multiple (contradictory) objectives have to be considered, e.g. maximising utilisation, minimising overtime and minimising waiting times, especially for infected patients, making the problems especially challenging. Another research question could be: If treatments must be postponed, how can they be scheduled after the crisis, when the incoming stream of new patients still exists and staff might already have performed much overtime during the pandemic?

### 3. Processes

A study with radiotherapy personnel could identify the time-consuming processes and processes with high workload. Then, it should be investigated how these could be reorganised to decrease workload and duration. A thorough analysis should be performed on the automation of processes in radiotherapy centres. Such an analysis should consistently consider all possibilities of automation independent of the short-term return on investment. The process analysis should also particularly consider the quality assurance processes in radiotherapy. Quality assurance in radiotherapy is legally required and a necessity to provide safe cancer care. A research question, also arising from topic 1, is how much radiotherapy processes could benefit from digitalisation. A simulation study could be used to analyse different chances, improvements and scenarios. Related to appointment planning for example, it could be investigated how long disinfecting linacs and rooms could reasonably take to keep as many appointments as possible and to minimise overtime.

### 4. Logistics for treating infected patients

Treating infected patients is especially challenging. When should their treatments be scheduled, how can cleaning and security measures be implemented with minimal overhead and costs and what are necessary resources? In addition, transportation of infected patients is challenging, if they cannot come by car themselves, but need an ambulance, for example.

### 5. After-crisis study

An after-crisis study can be conducted following the passing of the COVID-19 pandemic. The survey with participants would investigate the success of the different measures that were implemented in the subject matter context. For this purpose, all occupational groups in the radiotherapy centre should be interviewed. Also, available process data from the various radiotherapy systems should be evaluated. Furthermore, the impact on the overall risk management approach of radiotherapy centres could be analysed. Finally, the impact of the COVID-19 pandemic on policies and directives could be evaluated.

## Summary and conclusion

46.7% of the radiotherapy centres are affected by COVID-19. The centres experience longer process times due to protective measures and expect the situation to worsen. 75.8% expect restrictions due to non-availability of personnel. Measures have been implemented including information policy, access restrictions and increased hygiene. The highest COVID-19 impact is foreseen for the patient centric processes anamnesis, examinations, patient decision making, aftercare and appointment planning. Contingency plans are often based on two identical or technically compatible linacs (63.9%), but the two most implemented measures are non-technical: the classification of patients into urgency groups (41%) and treatment with hypofractionation schemes (33.7%). Radiotherapy centres would extend the applied measures in case of further dissemination of COVID-19. They would increasingly consider different options such as the application of additional fractions or replanning the overall therapy. Finally, radiotherapy centres would postpone or not treat patients with benign tumours and postpone aftercare examinations.

Most radiotherapy centres have implemented initial contingency measures. The measures are applied pragmatically, but the high risk of infection together with the associated risk of temporarily loss of personnel and ordered closure of business remains. The majority of radiotherapy centres in Germany sees a benefit in the integration of automated processes [37]. The COVID-19 crisis could lead to a breakthrough in digitalisation and related solutions in radiotherapy. At the submission of this paper on April 22nd the reported cumulative COVID-19 figures in Germany, Austria and Switzerland had reached 193.841 confirmed cases with 7.334 deaths and 134.528 recovered [38].

## Supporting information

S1 File Questionnaire download. (PDF)

S2 File Response data. (PDF)

S3 File COVID-19 news coverage during survey. (PDF)

S1 Dataset (XLSX)
