# Supplementary material for: Influence of the pandemic dissemination of COVID-19 on radiotherapy practice: A flash survey in Germany, Austria and Switzerland
Source: PLoS One. 2020 May 21;15(5):e0233330. doi: 10.1371/journal.pone.0233330 (PMC7241763; doi:10.1371/journal.pone.0233330)
Supplement: S1 File — (PDF) [file pone.0233330.s001.pdf]

Supporting information S2: questionnaire download

The questionnaire can be downloaded in English and German language at https://www.researchgate.net/project/Survey-on-the-impact-of-COVID-19-on-radiotherapy-centers-in-Germany-Austria-and-Switzerland
