# Supplementary material for: Influence of the pandemic dissemination of COVID-19 on radiotherapy practice: A flash survey in Germany, Austria and Switzerland
Source: PLoS One. 2020 May 21;15(5):e0233330. doi: 10.1371/journal.pone.0233330 (PMC7241763; doi:10.1371/journal.pone.0233330)
Supplement: S2 File — (PDF) [file pone.0233330.s002.pdf]

Supporting information S3: additional analysis

In the following, additional analysis of the response data is presented. The numbering follows the one used in the paper.

Table 2a. Number of patients treated per year.

n=43	average number of patients p.a.	min number of patients p.a.	max number of patients p.a.
ambulant patients	1946	130	3000
stationary patients	470	20	1800
benign tumours	662	20	2500
malign tumours	1726	440	3000

Table 3a. The processes in my radiation therapy are affected by COVID-19 linked to facility types.

n=112	Public hospital		Private hospital		Private centre		Other	
	Freq.	%	Freq.	%	Freq.	%	Freq.	%
Affected	21	**30.9**	2	22.2	15	**39.5**	0	0
Mainly affected	11	16.2	1	11.1	5	13.2	1	**50.0**
Partially affected	17	25.0	2	22.2	7	18.4	1	**50.0**
Mainly not affected	13	19.1	0	0	9	23.7	0	0
Not affected	5	7.4	1	11.1	1	2.6	0	0
Not specified	1	1.4	3	**33.3**	1	2.6	0	0

Table 3b. The processes in my radiation therapy are affected by COVID-19 linked to number of linacs.

n=112	1 linac		2 linacs		3 linacs		>4 linacs	
	Freq.	%	Freq.	%	Freq.	%	Freq.	%
Affected	2	18.2	15	**28.8**	8	**38.1**	13	**39.4**
Mainly affected	1	9.0	9	17.3	2	9.5	6	18.2
Partially affected	2	18.2	14	26.9	4	19.0	7	21.2
Mainly not affected	2	18.2	9	17.3	4	19.0	7	21.2
Not affected	0	0	5	9.6	2	9.5	0	0
Not specified	4	**36.4**	0	0	1	4.8	0	0

Table 4a. Processes in radiotherapy affected by COVID-19 linked to the facility types.

Answers (n=120, multiple selection)	Public hospital		Private hospital		Private centre		Other	
	Freq.	%	Freq.	%	Freq.	%	Freq.	%
Longer processes due to protective measures	39	**54.2**	3	50.0	22	**57.9**	1	25.0
Patients do not appear for appointments	24	33.3	4	**66.7**	22	**57.9**	1	25.0
Reduced own personnel	25	34.7	2	33.3	16	42.1	1	25.0
Limited access possibilities (traffic)	23	31.9	1	16.7	6	15.8	1	25.0
Supply chain affected	14	19.4	0	0	10	26.3	0	0
Reduced own personnel at co-handlers	10	13.9	0	0	4	10.5	0	0
Failure due to missing equipment service personnel	8	11.1	0	0	5	13.2	0	0

Table 6a. Restrictions expected in the coming weeks linked to facility types.

Answers (n=120, multiple selection)	Public hospital		Private hospital		Private centre		Other	
	Freq.	%	Freq.	%	Freq.	%	Freq.	%
Non-availability of own personnel	54	**75.0**	5	**83.3**	30	**78.9**	2	**50.0**
Longer processes due to protective measures	45	62.5	4	66.7	20	52.6	1	25.0
Patients do not keep appointments	39	54.2	4	66.7	23	60.5	2	**50.0**
Non-availability of personnel at co-handlers	30	41.7	3	50	18	47.4	2	**50.0**
Failure due to lack of access to service personnel	28	38.9	2	33.3	19	50	2	**50.0**
Supply chain affected	20	27.8	1	16.7	12	31.6	2	**50.0**
Limit access possibilities due to transport situation	24	33.3	1	16.7	7	18.4	1	25.0

Table 9a. Expected impact of COVID-19 on process steps of radiotherapy in public hospitals.

Answers (n=88)	Strongly agree (%)	Agree (%)	Unde-cided (%)	Disagree (%)	Strongly disagree (%)	Don't know (%)	N/A (%)
Appointment planning	**32.7**	30.9	21.8	5.5	1.8	0.0	7.3
Aftercare	29.1	**34.5**	12.7	5.5	1.8	3.6	12.7
Tumour conference	21.8	**30.9**	9.1	12.7	1.8	12.7	10.9
Anamnesis, examination, discussion	25.5	23.6	**29.1**	3.6	3.6	5.5	9.1
Patient decision making	12.7	**30.9**	21.8	10.9	1.8	7.3	14.5

Visits and final examination	9.1	**38.2**	27.3	7.3	3.6	5.5	9.1
Collection of patient data at admission	16.4	16.4	**25.5**	**25.5**	3.6	3.6	9.1
Imaging	5.5	20.0	20.0	**30.9**	10.9	5.5	7.3
Immobilisation for treatment	7.3	12.7	25.5	**27.3**	12.7	7.3	7.3
Patient verification	9.1	9.1	16.4	**27.3**	**27.3**	3.6	7.3
Treatment planning	1.8	9.1	23.6	**43.6**	14.5	1.8	5.5

Table 9b. Expected impact of COVID-19 on process steps of radiotherapy in private hospitals.

Answers (n=88)	Strongly agree (%)	Agree (%)	Unde-cided (%)	Disagree (%)	Strongly disagree (%)	Don't know (%)	N/A (%)
Appointment planning	**50.0**	0.0	16.7	16.7	16.7	0.0	0.0
Aftercare	**33.3**	**33.3**	16.7	0.0	0.0	16.7	0.0
Tumour conference	0.0	**50.0**	33.3	0.0	0.0	16.7	0.0
Anamnesis, examination, discussion	**50.0**	16.7	0.0	33.3	0.0	0.0	0.0
Patient decision making	0.0	33.3	**50.0**	16.7	0.0	0.0	0.0
Visits and final examination	16.7	0.0	**33.3**	**33.3**	0.0	16.7	0.0
Collection of patient data at admission	16.7	16.7	**33.3**	**33.3**	0.0	0.0	0.0
Imaging	0.0	16.7	0.0	33.3	**50.0**	0.0	0.0
Immobilisation for treatment	0.0	**33.3**	**33.3**	0.0	16.7	16.7	0.0
Patient verification	0.0	0.0	16.7	**66.7**	16.7	0.0	0.0
Treatment planning	0.0	0.0	0.0	**50.0**	**50.0**	0.0	0.0

Table 9c. Expected impact of COVID-19 on process steps of radiotherapy in private radiotherapy centres.

Answers (n=88)	Strongly agree (%)	Agree (%)	Unde-cided (%)	Disagree (%)	Strongly disagree (%)	Don't know (%)	N/A (%)
Appointment planning	**32.0**	28.0	**32.0**	4.0	0.0	0.0	4.0
Aftercare	20.0	**36.0**	8.0	12.0	0.0	16.0	8.0
Tumour conference	12.0	**40.0**	16.0	12.0	4.0	12.0	4.0
Anamnesis, examination, discussion	24.0	**32.0**	20.0	8.0	8.0	4.0	4.0
Patient decision making	4.0	**44.0**	20.0	12.0	4.0	12.0	4.0
Visits and final examination	0.0	**40.0**	16.0	24.0	0.0	12.0	8.0
Collection of patient data at admission	8.0	24.0	**36.0**	16.0	4.0	4.0	8.0
Imaging	0.0	12.0	20.0	16.0	**32.0**	8.0	12.0
Immobilisation for treatment	0.0	16.0	12.0	24.0	**36.0**	4.0	8.0
Patient verification	0.0	0.0	32.0	16.0	**36.0**	8.0	8.0
Treatment planning	0.0	12.0	12.0	24.0	**44.0**	0.0	8.0
