# Supplementary material for: Influence of the pandemic dissemination of COVID-19 on radiotherapy practice: A flash survey in Germany, Austria and Switzerland
Source: PLoS One. 2020 May 21;15(5):e0233330. doi: 10.1371/journal.pone.0233330 (PMC7241763; doi:10.1371/journal.pone.0233330)
Supplement: S3 File — (PDF) [file pone.0233330.s003.pdf]

Supporting information S4: COVID-19 news coverage during survey

The time frame of the survey was accompanied by dominant press communication on COVID-19 topics. Table 15 presents the daily news flow of the leading German news channel and the corresponding reported COVID-19 cases [39] [40]. ARD Tagesschau regularly reaches more than 10 million television viewers during prime time. Similar news have been communicated on most other channels in Germany.

Table 15. COVID-19 news during the time frame of the survey.

Wednesday, 25 March 2020 (42.400 reported COVID-19 cases)<br>18:57 Children in the Corona Crisis: "Patience, Perseverance and Time"<br>17:11 Corona crisis: federal government helps parents with loss of earnings<br>16:52 Buying hamsters: Through the crisis with toilet paper<br>15:55 Bundestag decides Corona aid package<br>02:41 Corona pandemic: "We are still at the beginning"<br>11:37 Doubts about the legality of measures against corona virus
Tuesday, 24 March 2020 (37.000 reported COVID-19 cases)<br>16:52 Economic crisis: Altmaier hopes for a rapid upswing<br>16:49 Coronavirus: Polish nurses stay at home<br>14:40 Corona is a particularly great danger for homeless people<br>13:01 Young Germans abroad: Return route cut off<br>03:47 - Coronavirus: "No vaccine is the greater risk"<br>02:07 Corona regulations: How the countries differ

Monday, 23 March 2020 (32.400 reported COVID-19 cases)<br><br>16:14 - Corona economic package: "Unprecedented" but "necessary<br><br>15:18 - Meuse to Corona retrieval: thousands of stranded people back home<br><br>15:05 - More money and less bureaucracy for hospitals<br><br>14:01 Corona crisis: Cabinet decides on billions in aid<br><br>07:46 - FAQ: What is in the Corona emergency package?
Sunday, 22 March 2020 (28.800 reported COVID-19 cases)<br><br>18:11 Merkel on corona measures: "No recommendations, but rules".<br><br>17:33 Live: Merkel on Corona measures<br><br>17:13 Churches in the Corona Crisis: When the Father comforts on YouTube<br><br>17:02 Agreement on comprehensive ban on contact<br><br>16:35 Live: Prime Ministers on the Corona Crisis<br><br>10:45 Corona: Experts recommend "Shutdown" until Easter<br><br>09:03 With Apps against the pandemic?<br><br>08:53 Consultations on Corona: Will the restrictions be tightened?<br><br>08:27 Corona measures: What you are still allowed to do - and what not<br><br>03:10 infection figures: Too early for a trend
Saturday, 21 March 2020 (26.500 reported COVID-19 cases)<br><br>22:26 Discussions on Corona: Are exit restrictions coming?<br><br>20:30 Corona crisis: Government puts together several aid packages<br><br>16:20 Corona crisis: Government plans protective measures for tenants<br><br>15:39 Government plans supplementary budget of 150 billion euros<br><br>13:48 Hardly any breaches of exit restriction

References

39. ARD. Tagesaktuelle Informationen zum Coronavirus; 2020 [Cited 2020 Mar 25]. Available from: https://www.bundesgesundheitsministerium.de/coronavirus.html#c17088.

40. Robert Koch-Institut: COVID-19-Dashboard; 2020 [Cited 2020 Mar 31]. Available from: https://experience.arcgis.com/experience/478220a4c454480e823b17327b2bf1d4.
